# Chronic ∆-9-tetrahydrocannabinol administration delays acquisition of schedule-induced drinking in rats and retains long-lasting effects

**DOI:** 10.1007/s00213-021-05952-2

**Published:** 2021-08-26

**Authors:** Esmeralda Fuentes-Verdugo, Gabriela E. López-Tolsa, Ricardo Pellón, Miguel Miguéns

**Affiliations:** 1 Departamento de Psicología Básica I, Facultad de Psicología, Universidad Nacional de Educación a Distancia (UNED), C/ Juan del Rosal 10, Ciudad Universitaria, 28040 Madrid, Spain; 2 Facultad de Psicología, Universidad Nacional Autónoma de México (UNAM), Av. Universidad 3004, Col. Copilco – Universidad, 04510 Ciudad Universitaria, Mexico City, Mexico

**Keywords:** THC, Cannabis, Adjunctive behaviour, Sensitization, Time estimation

## Abstract

**Rationale:**

Schedule-induced drinking (SID) is a behavioural phenomenon characterized by an excessive and repetitive drinking pattern with a distinctive temporal distribution that has been proposed as a robust and replicable animal model of compulsivity. Despite cannabis currently being the most widely consumed illicit drug, with growing interest in its clinical applications, little is known about the effects of ∆-9-tetrahydrocannabinol (THC) on SID.

**Objectives:**

The effects of chronic and acute THC administration on SID acquisition, maintenance and extinction were studied, as were the effects of such administrations on the distinctive temporal distribution pattern of SID.

**Methods:**

THC (5 mg/kg i.p.), or the corresponding vehicle, was administered to adult Wistar rats for 14 days in a row. Subsequently, THC effects on SID acquisition were tested during 21 sessions using a 1-h fixed-time 60-s food delivery schedule. Acute effects of THC were also evaluated after SID development. Finally, two extinction sessions were conducted to assess behavioural persistence.

**Results:**

The results showed that previous chronic THC treatment delayed SID acquisition and altered the distinctive behavioural temporal distribution pattern during sessions. Moreover, acute THC administration after SID development decreased SID performance in animals chronically pre-treated with the drug. No great persistence effects were observed during extinction in animals pre-treated with THC.

**Conclusions:**

These results suggest that chronic THC affects SID development, confirming that it can disrupt learning, possibly causing alterations in time estimation, and also leads to animals being sensitized when they are re-exposed to the drug after long periods without drug exposure.

**Supplementary Information:**

The online version contains supplementary material available at 10.1007/s00213-021-05952-2.

## Introduction

Cannabis plant derivatives are the most widely used illegal substances with the percentage of users per year estimated to be 3.8% worldwide and 5.2% in Europe (around 180 and 28 million users, respectively, aged 15–64 years), according to the World Drug Report of United Nations Offices on Drugs and Crime (2017). ∆-9-Tetrahydrocannabinol (THC) is the main component responsible for the psychoactive effects of cannabis. The harmful effects of prolonged THC consumption on both brain and behaviour—particularly when such consumption starts at an early developmental stage—are well documented in human and animal studies (for reviews, see Higuera-Matas et al. 2015; Volkow et al. 2016). The psychoactive properties of THC are mostly mediated by the activation of the type 1 cannabinoid receptor, which is expressed by different neuronal subpopulations in the central nervous system but also in peripheral tissues (Devane et al. 1992; Matsuda et al. 1990). THC acts on different aspects of behaviour such as learning, memory, motor activity, nociception and food intake (Calabrese and Rubio-Casillas 2018; Irimia et al. 2015; Iversen 2003; Javadi-Paydar et al. 2018). Another documented effect is the alteration of time perception. In this regard, it has been reported that cannabinoid users consistently overestimate the duration of time intervals (Lieving et al. 2006; Perez-Reyes et al. 1991; Sewell et al. 2013). This alteration in time estimation was also reported in non-human subjects (Conrad et al. 1972; Crystal et al. 2003; Han and Robinson 2001). In addition, it has been reported that some of the THC effects can be sensitized after repeated drug administration, as has the possibility that THC causes cross-sensitization when animals are exposed to other substances, which has led to the suggestion that cannabis can facilitate the use of other drugs of abuse (Cadoni et al. 2001; Panlilio et al. 2013).

Schedule-induced drinking (SID) is characterized by the development of repetitive excessive drinking in food-deprived animals that are exposed to intermittent food-reinforcement schedules with free access to a bottle of water in the experimental chamber. Once SID was characterized, it was included in an extensive behavioural category called adjunctive behaviour (Falk 1971; 1977) and it is well documented that SID presents a distinctive temporal pattern where most drinking occurs early in the inter-food interval, just after food delivery (Falk 1971; López-Crespo et al. 2004; Staddon 1977). In this respect, it has been suggested that adjunctive behaviours could play an important role in time estimation (Harper and Bizo 2000; Killeen et al. 1997) operating as a behavioural clock through collateral behaviour chains that precede each other until reinforcer presentation occurs (Lejeune et al. 2006; Richelle and Lejeune 1984; Richelle et al. 2013). In addition, SID could also serve as a cue for organisms to discriminate time (Killeen and Fetterman 1988), and it may be that in this way SID expedites the learning of different time estimation tasks (Ruiz et al. 2016; Segal and Holloway 1963).

The excessiveness and persistence of SID may share common features with compulsive behaviour in humans, and for this reason, it has been proposed as a useful and validated animal model to study several disorders related to the compulsive spectrum (Moreno and Flores 2012; Woods et al. 1993). Alterations in neural substrates involved in the development and execution of habits contribute to compulsive behaviour (Fineberg et al. 2010; Gillan et al. 2016). Recent studies have suggested increased habit formation in rats with high drinking rates (Merchán et al. 2019). Moreover, rats with a preference for response-learning strategies are more susceptible to developing SID and show increased neuronal activation in frontal cortical regions associated with habit formation and compulsion (Gregory et al. 2015). Furthermore, in our laboratory, we have reported that SID is associated with increased dendritic spine density in dorsolateral striatum neurons (Íbias et al. 2015)—a region that appears to be involved in habit formation (Yin et al. 2004; 2006). It is interesting to note that cannabinoids are also involved in the transition from volitional behaviour to habit formation and they induce structural plasticity alterations in regions related to this kind of behaviour (Goodman and Packard 2015).

The studies that consider SID as a model of compulsivity focus on testing the efficacy of drugs normally used to treat the symptoms of different disorders (Moreno and Flores 2012), including obsessive–compulsive disorder (OCD; Platt et al. 2008), mood disorders (Martin et al. 1998; Rosenzweig-Lipson et al. 2007; Woods et al. 1993), anxiety (Snodgrass and Allen 1989), schizophrenia (Hawken and Beninger 2014), and ADHD (Íbias et al. 2016). For example, benzodiazepine agonists (Mittleman et al. 1988), and different types of antipsychotics, decreased SID after their acquisition (Didriksen et al. 1993; Snodgrass and Allen 1989; Todd et al. 1992); antidepressants (Martin et al. 1998; Rosenzweig-Lipson et al. 2007; Woods et al. 1993) produced a dose-dependent reduction (Dwyer et al. 2010); and dopamine agents, such as methylphenidate and d-amphetamine, also reduce SID behaviour in a dose-dependent manner although observations varied according to the rat strain (Íbias et al. 2016).

Furthermore, some psychoactive recreational drugs were tested for their effects on SID. Amphetamines have been used to study their differential effects on operant and adjunctive behaviours (Flores and Pellón 1995; 1997; Pellón et al. 1992; Smith and Clark 1975; Wayner et al. 1973b). Scopolamine and high doses of methamphetamine dose-dependently reduced compulsive drinking, but no relevant effects were found using ketamine, AM404 or the cannabinoids cannabidiol and WIN 55,212–2 (Martín-González et al. 2018). It should be noted that one previous study did test the effects of THC on SID, showing that THC enhanced drinking behaviour; however, this study was limited both by the small number of animals evaluated, as by the doses employed, which were too low compared to those associated with recreational or therapeutic use (Wayner et al. 1973a). Moreover, it only assessed acute effects of THC on SID, while it is more common in cannabinoids users to develop complications due to habitual consumption (Leung et al. 2020). Thus, the interest of this work was to study acute and chronic THC effects on SID.

Finally, it is important to consider that despite the illicit status and harmful effects of cannabinoids, there is growing interest in their therapeutic use in several psychiatric disorders, such as post-traumatic stress disorder, anxiety, depression and—of particular relevance to compulsivity—Tourette syndrome (Curtis et al. 2009; Fraser 2009; Moreira et al. 2009; Robson 2001; Tambaro and Bortolato 2012). Additionally, clinical cases with OCD who are also cannabis users report that it improves their symptoms (Müller-Vahl 2013; Schindler et al. 2008). In this regard, it was recently suggested that the administration of nabilone, a synthetic cannabinoid that mimics THC effects, in combination with exposure and response prevention therapy, resulted in a significant decrease in OCD severity (Patel et al. 2021). All of these findings have led to the endocannabinoid system (ECS) being considered a target for novel medications for OCD symptoms (reviewed in Kayser et al. 2019), and the SID procedure provides the opportunity to assess the effects of THC on a behaviour validated as an animal model of compulsivity.

In the present study, the effects of chronic and acute THC administration on SID acquisition and maintenance were evaluated. The effects of such administrations on the distinctive temporal distribution pattern of SID were also studied.

## Materials and methods

### Subjects

A total of 20 naïve male Wistar rats obtained from Charles River Laboratories (Lyon, France) were used in these experiments. On arrival, the rats were 8 weeks old. They were initially housed in groups of four, and once habituated to the animal facility for a week, the animals were singly housed in transparent polycarbonate cages (18 cm × 32 cm × 20.5 cm) with a metal grid roof, and food and water freely available. The room environment was maintained with a 12-h light/12-h dark cycle (light from 8:00 to 20:00 h), an ambient temperature of 20 ± 2 °C, and approximately 55% relative humidity. Ten of these rats were randomly assigned to the group treated with THC (THC group), while the other 10 served as vehicle controls (vehicle group).

At the start of the experiment, the animals were 12 weeks old, and their mean (± SEM) weights were 369 ± 17 g and 369 ± 20 g for the vehicle group and THC group, respectively. Animal weights were maintained by controlled feeding to 100% of their free-feeding body weights with reference to a standard growth curve for the Wistar strain during the chronic THC (or vehicle) treatment phase but were then gradually reduced to 85% before starting the SID acquisition procedure. This reduced weight was maintained by controlled dieting throughout the different experimental phases of the study. The rats were weighed daily before the experimental sessions and fed at least 20 min after their completion. All animal care procedures were conformed to the European Union Council Directive 2010/6 and the Spanish Royal Decree 53/2013 for minimizing stress and discomfort in animals with the corresponding authorization from the Community of Madrid (PROEX 077/18) and UNED bioethics committee.

### Drug preparation

THC was obtained from THC Pharm Gmbh (Frankfurt/Main, Germany) and was prepared daily in aliquots for a final concentration of 5 mg/ml, in a vehicle of absolute ethanol (Emsure Merck KGaA; Darmstadt, Germany), cremophor (KolliphorEL; Sigma Aldrich Co.; St. Louis, MO, USA) and saline (0.9% sodium chloride) in a ratio of 1:1:18. This ratio is commonly used for the solubilization of cannabinoids (Cha et al. 2006; Rubino et al. 2009). The ethanol concentration in the THC and vehicle solutions was 5%, resulting in ethanol doses of 0.02 g/kg. The drug was stored in an N_2_ atmosphere to avoid the oxidation process and was kept refrigerated (− 35 °C) in darkness until just prior to administration.

### Apparatus

Eight Letica Li-836 (Letica Instruments, Barcelona, Spain) conditioning chambers (29 cm × 24.5 cm × 35.5 cm) were used, each of which was enclosed inside a soundproof wooden box with a window on the front. The conditioning chambers walls were made of aluminium and polycarbonate. The right wall had an aperture (3.2 cm × 3.9 cm) where a bottle of water was set 7 cm above the grid floor. The contact between the spout of the bottle and the grid floor closed an electric circuit which allowed the automatic record of licks when an animal touched the spout with its tongue. A food dispenser delivered food pellets of 45 mg (Bio-Serv, Frenchtown, NJ, USA) in an aperture in the frontal aluminium wall situated 3.7 cm from the floor, between two levers, retracted during the experiment. Magazine entries were sensed by a photocell beam at the entrance of the aperture that provided access to the food magazine. The chambers had a 3-W lamp above each lever that remained off, and another 25-W light—installed in the interior of the soundproof boxes—that was kept on throughout the experimental sessions. A sawdust tray was placed under the grid floor. Inside the soundproof boxes, a ventilation system with a fan produced a background noise of 60 dB which masked the exterior noises. Licks and magazine entries were registered with a MED-PC-IV application in the operating system Windows 7.

### THC chronic administration

Chronic treatment with either THC (*n* = 10) or the corresponding vehicle (*n* = 10) lasted 14 days. During this period, rats received one daily intraperitoneal (i.p.) injection at the same time of day (15:00 h). Doses of 5 mg/ml were estimated for i.p. injections in a volume of 1 ml/kg body weight to give a final THC concentration of 5 mg/kg in the THC group, while an equivalent injection volume of the vehicle was administered to control rats. This dose was chosen because it has been demonstrated that behavioural effects normally appear at doses of 2 or 5 mg/kg (Fadda et al. 2004) and this level of dosing has been used before in chronic administration procedures (Cha et al. 2006). To rule out the presence of active metabolites and withdrawal effects after chronic THC treatment, a clearance period of 7 days was allowed before behavioural testing began.

### SID acquisition

Both groups of rats (THC pre-treated group and their vehicle controls, *n* = 10 in each) were subjected to a fixed-time (FT) schedule in which food pellets were regularly delivered regardless of animal behaviour to develop SID. A total of 21 daily sessions were conducted to study SID acquisition and maintenance. The fixed time for food delivery was 60 s and each session lasted 1 h. A bottle of water was available in the conditioning chambers over the course of the experimental sessions.

### Acute THC administration tested on SID

The acute THC administration test was divided into two experimental sessions after SID acquisition and stable baselines of drinking were established. The first session consisted of a control session where an i.p. injection of the vehicle solution was administered to both groups to rule out indirect effects on behavioural testing. In the second session, all animals were i.p. injected with THC (5 mg/kg in a volume of 1 ml/kg body weight) to assess acute effects on SID in rats previously treated chronically with THC and in rats that had no prior exposure to the drug. In both sessions, the rats received one i.p. injection 1 h before starting in the conditioning chambers, following the same procedure of SID as in the acquisition phase.

After a clearance period of 7 days, five sessions with the same fixed-time food schedule were carried out to restore SID behaviour to a stable drinking level.

### SID extinction

The extinction test was conducted in two sessions of 1 h each, but the food pellets were removed from the food dispenser before the start of the session. The food dispenser worked according to the same FT-60 s schedule as in previous phases, which produced a clicking sound not accompanied by delivery of pellets. A bottle containing water was also available in the conditioning chambers.

**Fig. 1:**
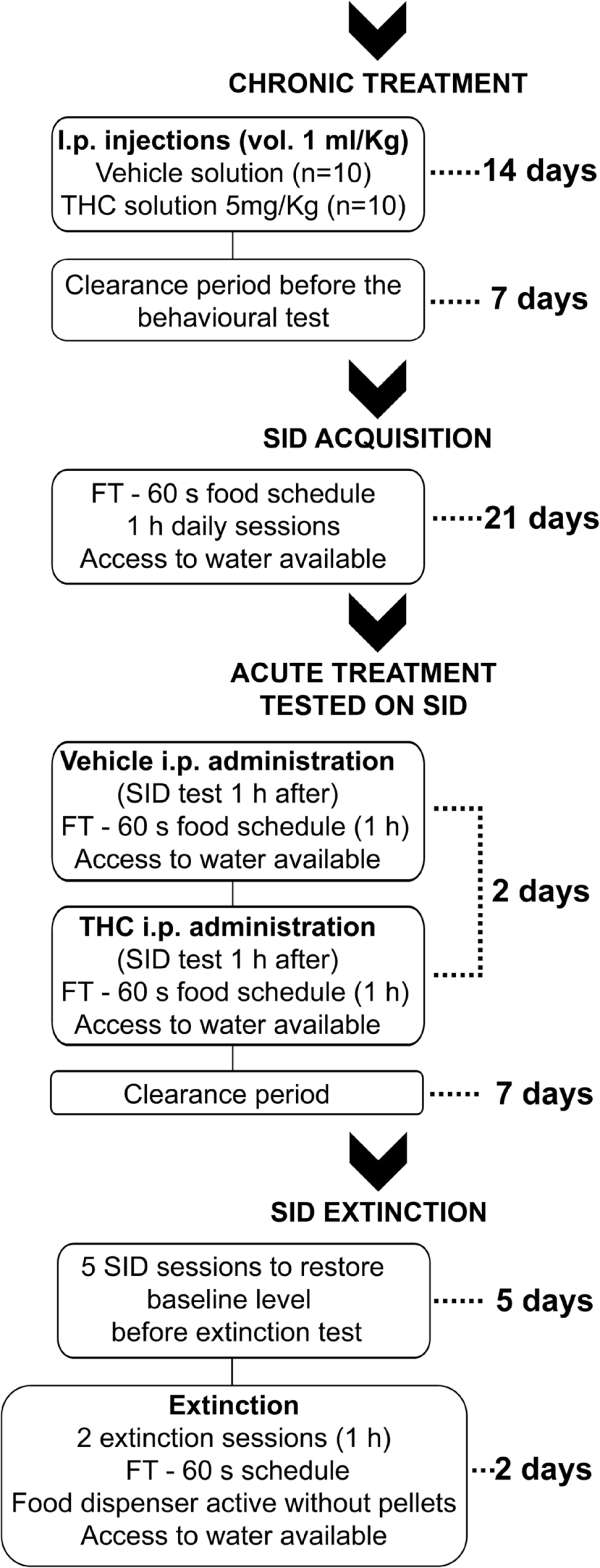
Timeline of the different experimental phases of the study

The sequence and timing in each experimental phase is illustrated in Fig. 1.

### Statistical analysis

**Fig. 2:**
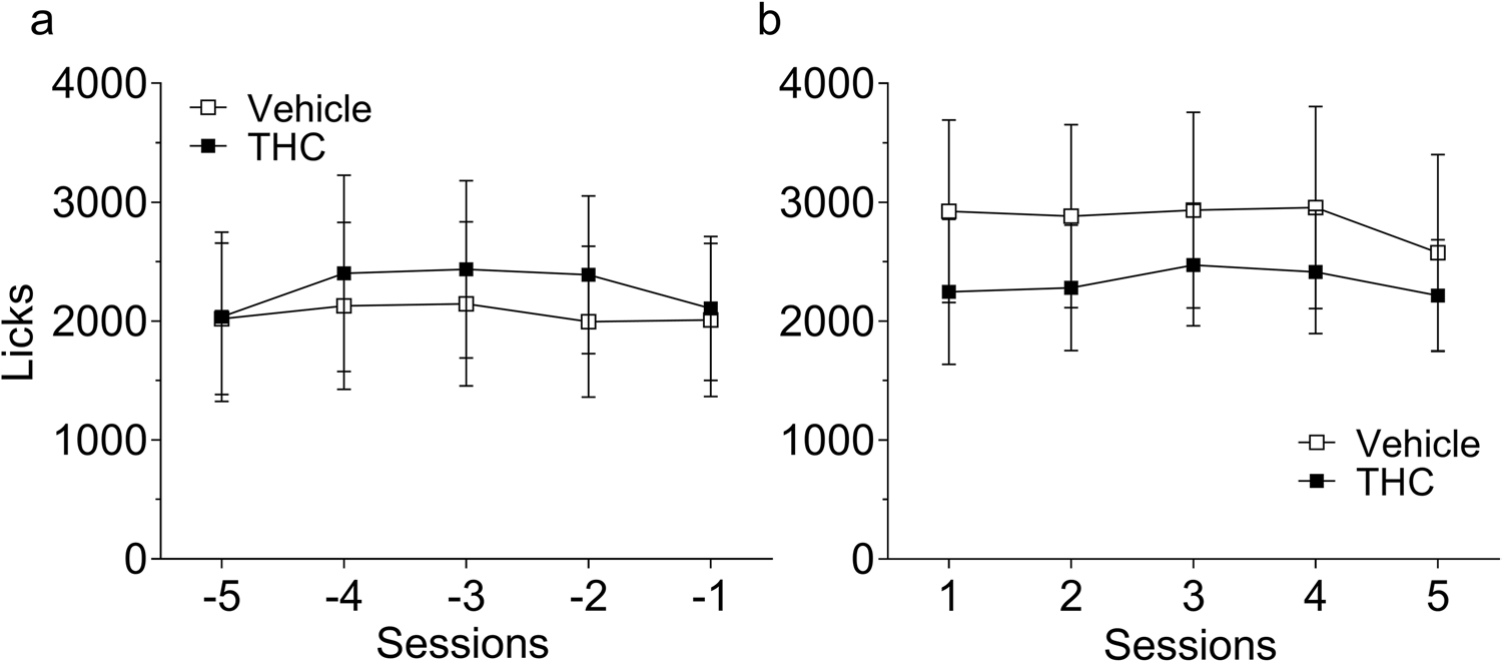
Total number of licks after SID acquisition and maintenance. Total number of licks (mean ± SEM) for vehicle (white squares, *n* = 9) or THC pre-treated animals (black squares, *n* = 9) during the last five acquisition sessions, established as the baseline to transform the data in acquisition and drug test phases (**a**), and during the five sessions conducted before extinction used as the baseline to transform the data of this phase (**b**)

A data normalization was carried out prior to the analysis. The total number of licks was transformed into a percentage for each subject with respect to the average of the last five acquisition sessions (Fig. 2a), both for acquisition and acute drug administration. For extinction, data were normalized to the average of the five sessions conducted prior to extinction (Fig. 2b). During these sessions, the rats reached an asymptotic and stable level of licking and therefore, their mean was used as a reference to calculate the percentage of change. Animals with less than 250 licks during the last 5 sessions did not develop the characteristic temporal distribution of licking normally observed in SID procedures, and were removed from the analysis (this was the case for one subject for each group, leaving final groups of *n* = 9). Magazine entries were also recorded and analyzed, but no significant differences were found (for more information, see supplementary material).

Analyses were conducted using IBM SPSS statistical software package (version 24 for Windows). The differences in percentage of licks in all the phases between the vehicle and THC groups were analyzed using a mixed analysis of variance (ANOVAs) with the between-subjects factor ‘*pre-treatment*’ with two levels, vehicle and THC, and the within-subjects repeated measures factor ‘*sessions*’ with one level for each experimental session. Post hoc comparisons were carried out using pairwise comparisons with Bonferroni correction, with statistically significant *p* values of *α* < 0.05. Effect size was calculated using partial eta squared (*η*^2^_*p*_). Sphericity principle violations were evaluated with the Mauchly Sphericity test and significant deviations from this principle were corrected using Greenhouse–Geisser (GG) epsilon (*ε*) to adjust the degrees of freedom with *α* = 0.05. SID temporal distribution was studied through the descriptive parameters obtained with the area under the curve using the trapezoidal rule; these are the highest point of the *x*-axis which represents the peak time, the highest point of the *y*-axis which represents the peak percentage of licks, and the total area of the temporal distribution in function of time and percentage of licks.

## Results

### Chronic THC administration delayed SID acquisition

**Fig. 3:**
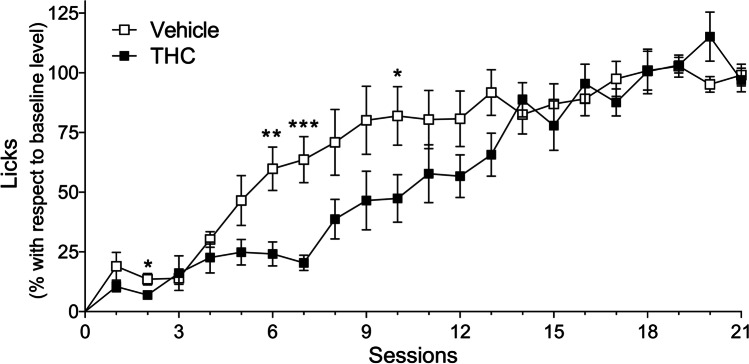
Chronic THC administration delayed SID acquisition. Percentage of licks with respect to the average level reached during the last five sessions of the acquisition phase. The percentage of licks is represented over the course of 21 SID sessions (mean ± SEM). White squares represent vehicle pre-treated rats (*n* = 9); black squares represent THC (mg/kg) pre-treated rats (*n* = 9). ***p < 0.001, **p < 0.01, *p < 0.05 using Bonferroni post-test

Lick acquisition curves for the group pre-treated with THC and its vehicle control are represented in Fig. 3. The data (mean ± SEM) are shown as percentages with respect to the 5 last sessions (Fig. 2a) over the course of 21 sessions. The mixed ANOVA revealed statistically significant effects for both *session* (*F*_4,69_ = 37.326; GG (*ε*) = 0.217; *p* < 0.0001; *η*^2^_*p*_ = 0.7) and *pre-treatment* (*F*_1,16_ = 4.632; *p* < 0.05; *η*^2^_*p*_ = 0.224). These results indicated, firstly, that the exposure to the fixed-time 60-s food schedule increased the licks over the course of the acquisition sessions and, secondly, that the rats pre-treated with THC licked fewer than their vehicle controls. Moreover, the statistically significant *pre-treatment* x *session* interaction effect (*F*_4,69_ = 2.779; *p* < 0.05; *η*^2^_*p*_ = 0.148) revealed after post hoc analysis that SID acquisition was delayed in the rats pre-treated with THC due to an effect of the drug in the sessions after the very initial ones (sessions with post hoc differences shown in Fig. 3).

### Acute THC administration reduced SID in THC but not in vehicle pre-treated animals

**Fig. 4:**
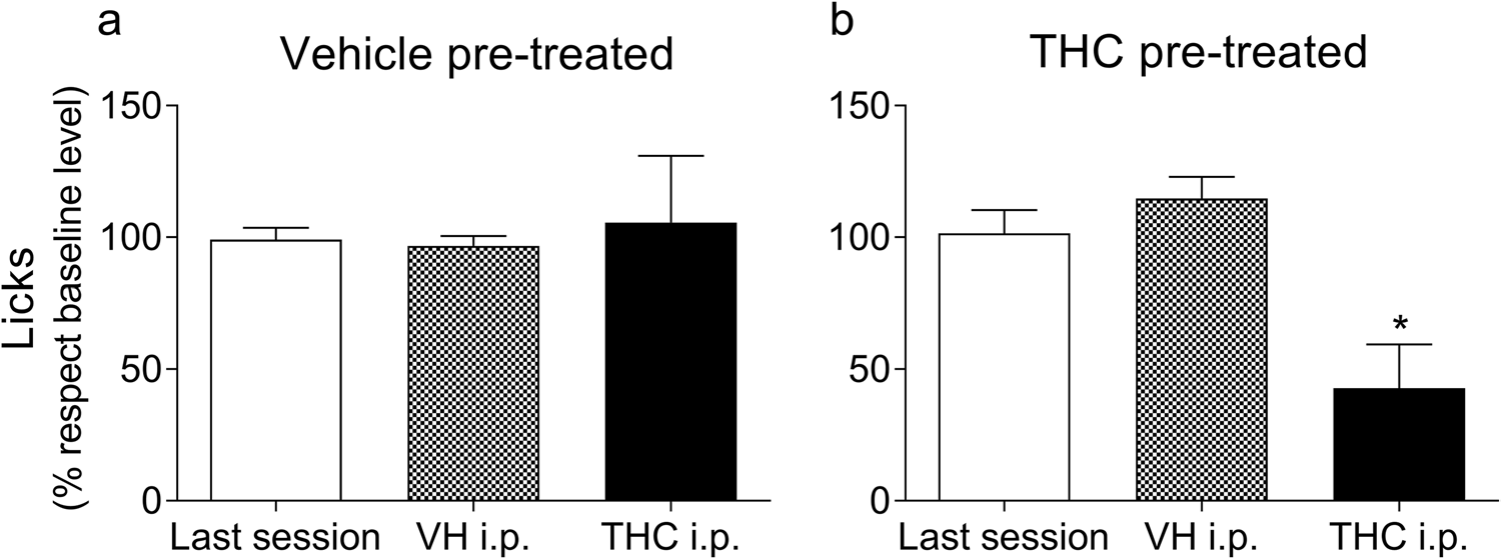
Acute THC administration (5 mg/kg i.p.) reduced SID only in animals previously pre-treated with THC. The percentage of licks with respect to the previous five acquisition sessions (baseline level) is represented in the group pre-treated with vehicle (**a**) and the group pre-treated with THC (**b**). The data represent the mean ± SEM in the last session, the control session with a preceding vehicle i.p. injection, and the test session with a preceding THC i.p. injection (*n* = 9 in each group). *p < 0.05 using Bonferroni post-test

Figure 4 shows lick percentage (mean ± SEM) with respect to the mean of the last five acquisition sessions (Fig. 2a), as indicated previously, in animals that were chronically pre-treated with vehicle (Fig. 4a) and in animals that were chronically pre-treated with THC (Fig. 4b). These figures include data for the last acquisition session, the session where all animals were i.p. injected with the vehicle solution, and the test session where all animals were i.p. injected with a single 5-mg/kg dose of THC. The ANOVA revealed a statistically significant *session* effect (*F*_1,19_ = 8.61; GG (*ε*) = 0.607; *p* < 0.01; *η*^2^_*p*_ = 0.843) and a *pre-treatment* x *session* interaction effect (*F*_1,19_ = 5.201; *p* < 0.05; *η*^2^_*p*_ = 0.631). No main effects were found in the between-subjects factor *pre-treatment* (*F*_1,16_ = 390.15; *p* = 0.283, ns; *η*^2^_*p*_ = 0.072). Acute i.p. administration of the vehicle did not modify the lick percentage in any group, confirming that there are no indirect vehicle or injection-related effects. However, as indicated by the statistically significant effects for *session* and the *pre-treatment* x *session* interaction, acute i.p. administration of THC resulted in a reduction in licking in animals pre-treated with THC shown by post hoc analysis (*p* < 0.05), but this was not the case with the vehicle. The main effect in the between-subjects factor *pre-treatment* did not show differences between groups during the session which THC was administered, but an increased variability in drinking behaviour in animals that had not had prior exposure to the drug was observed as a result of the acute effect of THC. Subsequent descriptive analysis of this variability revealed an increased percentage of the SD—from ± 13.67% obtained in the last acquisition session or ± 11.25% obtained in the control session with vehicle, to ± 76.17% as a result of the effects of THC in this group.

### Chronic THC administration had no statistically significant effects on SID extinction

**Fig. 5:**
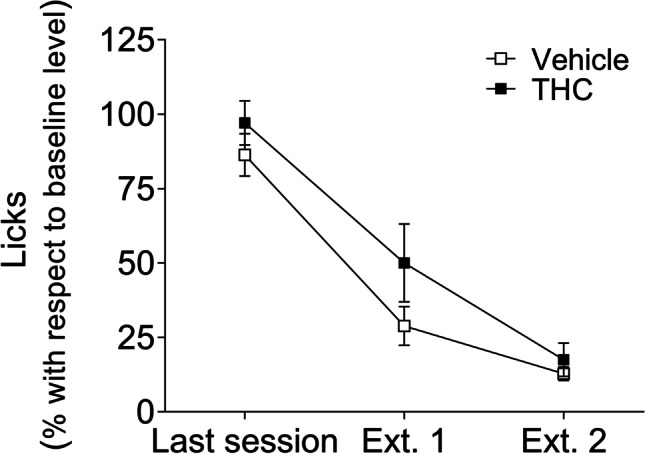
THC did not affect behavioural performance during SID extinction. Percentage of licks with respect to baseline level (mean ± SEM) in the two SID extinction sessions for vehicle and THC pre-treated animals (*n* = 9 in each group)

Figure 5 shows the results obtained in SID extinction sessions in vehicle and THC-pre-treated rats. Data (mean ± SEM) are represented as the percentage of licks with respect to the mean of the five previous SID sessions (Fig. 2b). The ANOVA revealed a statistically significant session effect (*F*_2,32_ = 52.575; *p* < 0.0001; *η*^2^_*p*_ = 0.767) observed between the last session and the extinction sessions. No statistically significant effects were found for *pre-treatment* (*F*_1,16_ = 227.293; *p* = 0.078, ns; *η*^2^_*p*_ = 0.182) or for *session* x *pre-treatment* interaction (*F*_2,32_ = 0.599; *p* = 0.599, ns; *η*^2^_*p*_ = 0.555). These results show that the simple intermittent activation of the dispenser without delivering food pellets reduced SID in both groups at a similar rate.

### Chronic and acute effects of THC on the temporal distribution of SID

#### Prolonged temporal distribution of SID after prior chronic THC exposure

**Fig. 6:**
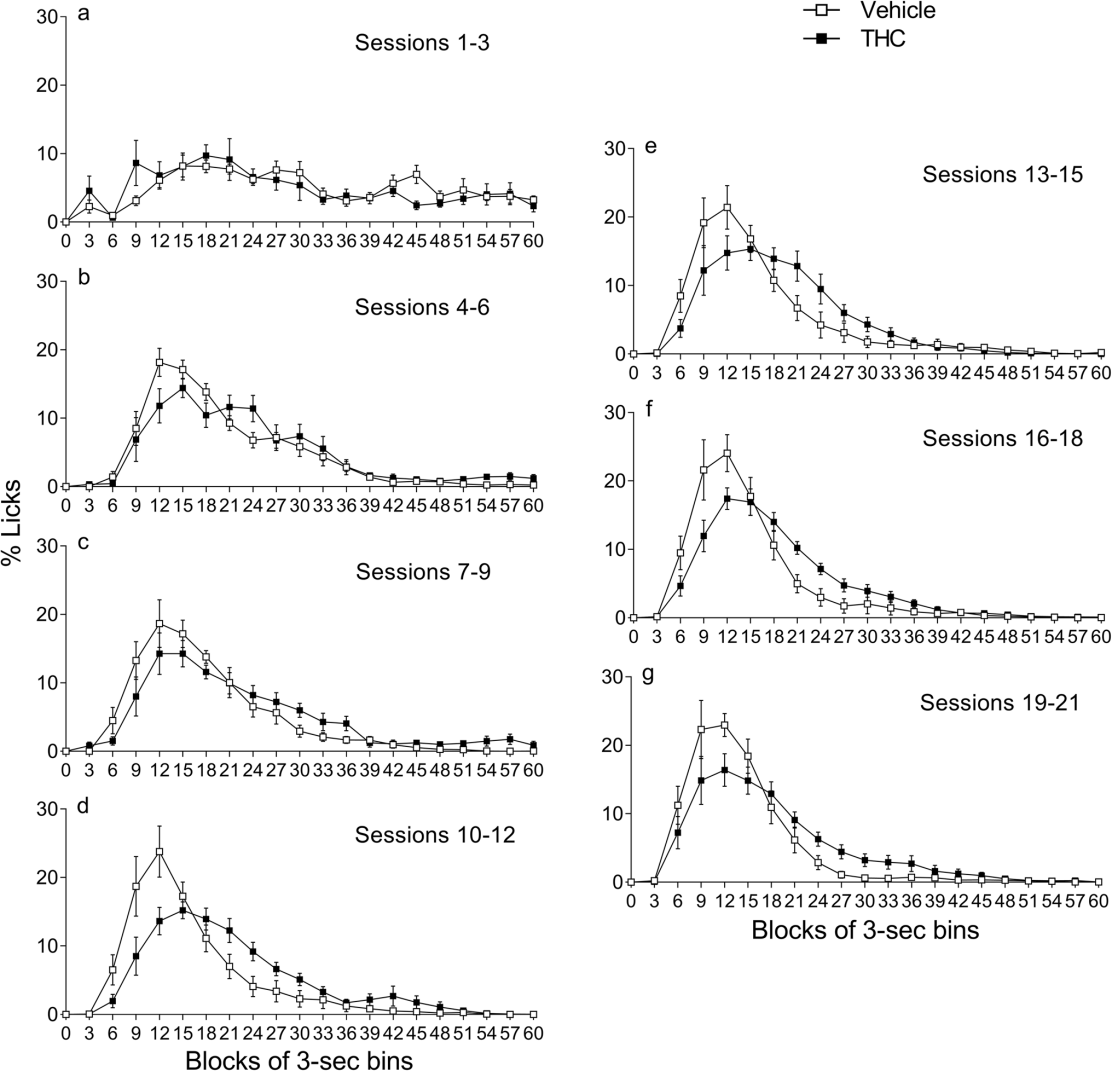
Chronic effects of THC on the temporal distribution of licking throughout SID acquisition sessions. Percentage of licks with respect to the total number of licks performed in the inter-food interval for each rat (mean ± SEM) throughout successive 3-s bins in animals pre-treated with vehicle or THC (*n* = 9 in each group). Separate sets of 3 sessions are presented to show acquisition development

Figure 6 displays THC pre-treatment effects on licking behaviour—compared with the vehicle controls—in successive 3-s bins during the inter-food interval (60 s) in separate sets of 3 sessions (Fig. 6a to g) to observe temporal SID distribution features over the course of acquisition. The data were calculated as percentages (mean ± SEM) with respect to the total number of licks performed in the inter-food interval for each rat. The parameters studied with the area under the curve were the highest point of the *x*-axis (peak time), the highest point of the *y*-axis (peak percentage of licks) and the total area of the temporal distribution as a function of time and percentage of licks (data in Table 1).

**Table 1:** Descriptive parameters obtained with the area under the curve of the temporal distribution of licks over the course of SID acquisition

Sessions	Peak time (s)		Peak licks (%)		Total area (% ± SEM)	
	Vehicle	THC	Vehicle	THC	Vehicle	THC
Ss.1–3	15	18	8.2	9.7	295.2 ± 29.4	296.4 ± 46.6
Ss.4–6	12	15	18.2	14.4	299.6 ± 32.3	298.2 ± 39.3
Ss.7–9	12	12	18.7	14.3	300.0 ± 40.3	298.7 ± 37.7
Ss.10–12	12	15	23.8	15.2	299.8 ± 48.3	300.0 ± 34.2
Ss.13–15	12	15	21.4	15.3	299.7 ± 43.6	300.0 ± 40.5
Ss.16–18	12	12	24.1	17.4	299.9 ± 46.6	299.8 ± 28.7
Ss.19–21	12	12	22.9	16.4	299.9 ± 43.2	300.0 ± 39.7

During the first 3 sessions, neither of the two groups showed the distinctive post-pellet drinking pattern of SID (Fig. 6a); the licks remained similar throughout the entire inter-food interval. From sessions 4 to 21 (Fig. 6b to g), the temporal distribution of licking progressively acquired the typical SID pattern, showing a maximal response close to the previous food delivery in both groups. In sessions 4–6 (Fig. 6b), 10–12 (Fig. 6c) and 13–15 (Fig. 6d), the peak time was lower and shifted 3 s to the right in THC-pre-treated animals compared to the vehicle group (Table 1). Although the THC group showed a lower peak for percentage of licks, the duration of their licking was longer (from second 18 onwards; Fig. 6d to g). Thus, during the last acquisition sessions, THC-pre-treated animals showed similar total licking levels overall, but with a different response pattern.

#### Non-temporal distribution pattern found during extinction

The reduction of licks in the extinction phase occurred rapidly in both groups and there was no sign of the characteristic temporal pattern of SID, which is why no data are presented here.

#### Acute effects of THC in the temporal distribution of SID are dependent on previous drug exposure

**Fig. 7:**
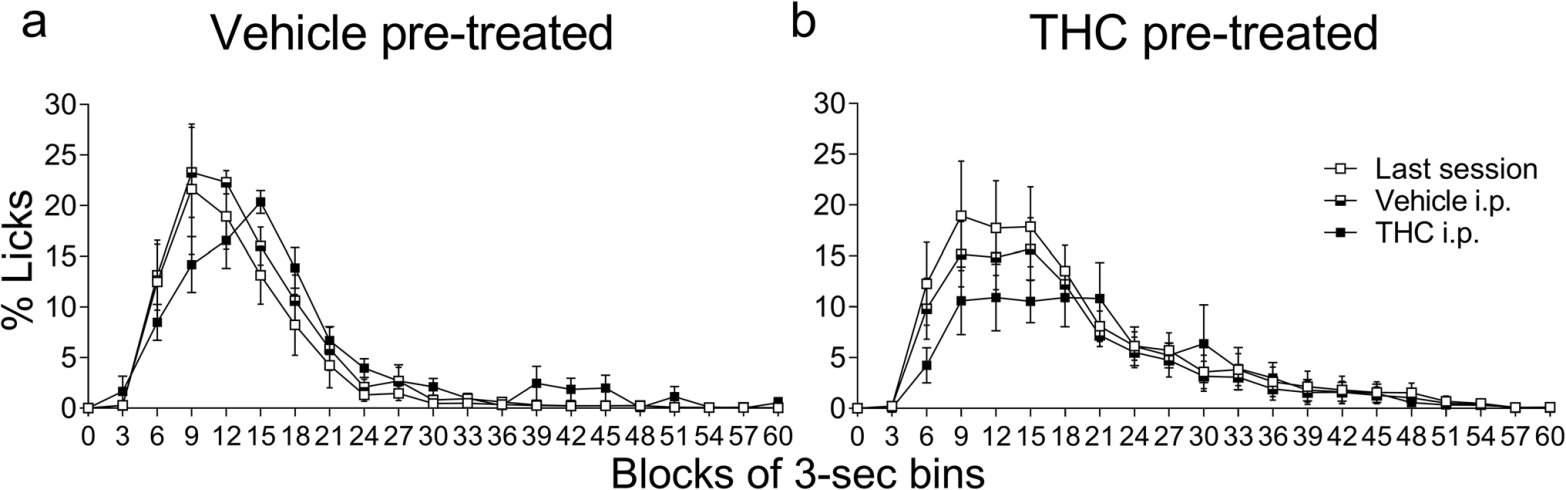
Acute effects of THC on the temporal distribution of licking in animals pre-treated with THC or vehicle. The data show the percentage of licks with respect to the total number of licks performed in the inter-food interval (mean ± SEM) in animals pre-treated with THC (**a**) or vehicle (**b**) throughout successive 3-s bins of the 60-s inter-food interval during the last acquisition session, the control session with a preceding vehicle i.p. injection, and the test session with a preceding THC i.p. injection (*n* = 9 in each group)

Figure 7 displays the acute effects of THC administration on the temporal distribution of licking over the course of the inter-food interval (60 s) in animals chronically pre-treated with vehicle (Fig. 7a) and in animals previously pre-treated with THC (Fig. 7b). It shows the data of the last acquisition session, the session where all animals were i.p. treated with the vehicle solution, and the acute THC administration test session using a 5-mg/kg dose of THC in both groups. The data showed that the peak time in animals pre-treated with vehicle was 6 s later (Fig. 7a and Table 2), comparing the results of the acute THC administration test (peak time in 15 s) with the last acquisition session and the vehicle control session (with peak times reached around the second 9 in both cases). The peak percentage of licks was similar during the three sessions in this group (Table 2), which is consistent with the results shown in Fig. 3. Figure 7b shows the results of animals pre-treated with THC. The percentage of licks was considerably lower in this group (Fig. 4), but their temporal distribution did not show any appreciable change. Peak times occurred at different time points compared with those in control sessions (Table 2), but not markedly different in that the peak of the curve was maintained from seconds 9 to 21 with a similar percentage of licks (Fig. 7b). The data showed that the most affected parameter was the kurtosis of the curve, but not the symmetry. The peak of the percentage of licks remained at lower levels compared with control sessions and the same occurred with the total area (Fig. 7b and Table 2), which was consistent with the reduction of licks shown on the test day with THC (see Fig. 4).

**Table 2:** Descriptive parameters obtained with the area under the curve of the temporal distribution of licks in SID during THC acute test

	Vehicle pre-treated			THC pre-treated		
	Last session	Vehicle i.p	THC i.p	Last session	Vehicle i.p	THC i.p
Peak time (s)	9	9	15	9	15	12
Peak licks (%)	21.6	23.3	20.4	18.95	15.7	10.9
Total area (% ± SEM)	252.7 ± 60.2	299.9 ± 45.2	299.1 ± 39.8	356.6 ± 66.9	299.8 ± 48	266.6 ± 56.8

## Discussion

The results of the present study showed that chronic THC administration (5 mg/kg for 14 days) delayed SID acquisition and resulted in a flattening and shifting to the right of licking behaviour temporal distribution over the course of the sessions. Moreover, acute THC administration after SID acquisition resulted in an overall decrease in licking only in animals that were previously chronically treated with THC, pointing to a sensitization effect. However, no significant THC-related effects were observed during SID extinction.

The number of magazine entries did not show significant differences among groups—either during SID acquisition after chronic pre-treatment with THC or as an acute effect of the drug before a SID session (see supplementary material), suggesting that THC effects on SID were not driven by general locomotor suppression or lack of motivation. Similar results with regard to magazine entries were also reported when the acute effects of WIN 55,212–2 were assessed (Martín-González et al. 2018). It is also important to note that motor suppression usually occurs at higher doses of cannabinoids (de Fonseca et al. 1998) than the 5 mg/kg used in the present study.

THC and vehicle chronically pre-treated animals developed SID over the course of 21 sessions of a FT 60-s food schedule (Fig. 3). Nevertheless, in the case of the THC-pre-treated animals, SID acquisition was delayed and developed more slowly, requiring 14 sessions to reach the same level as the vehicle control group, which acquired SID earlier (from session 4 onwards). The reason for this delay could be associated with the fact that THC pre-treatment alters learning mechanisms involved in the performance of SID as well as in the experimental tasks that follow. The acquisition of the reinforcement of low-rate responding task was delayed in rats chronically pre-treated with THC (Stiglick and Kalant 1982; 1983). Moreover, as occurred in our study, control and THC-pre-treated animals reached similar levels at the end of the acquisition phase. In the aforementioned studies, rats were treated firstly in adolescence, and when the procedure was replicated in adult rats, no effects were found, suggesting the existence of vulnerable periods during which THC impairs learning (Scallet 1991; Stiglick and Kalant 1985). A worsening performance was also observed in the object recognition task or in progressive ratio reinforcement schedules in adolescent—but not in adult—rats pre-treated with the synthetic cannabinoid agonist CP 55,940 (O’shea et al. 2004; Schneider and Koch 2003). The relevance of THC administration during sensitive developmental periods has been documented in studies where rats, which were exposed to cannabinoids before being born, in perinatal periods, or during adolescence, showed later alterations in learning and memory in adulthood (Campolongo et al. 2007; Rubino et al. 2009). However, our results showed that the effects of a previous chronic THC administration can also alter learning in adult subjects. Even so, it would be necessary to determine if there are differential effects on the acquisition of SID when THC is administered during vulnerable periods of development.

Once SID was established (from sessions 10 to 14 until the last session), the temporal distribution of licks in animals that were chronically pre-treated with THC showed lower peaks of licks percentage than those of the vehicle control group (see Fig. 6 and Table 1). If we compare the peaks observed in these sessions with the results obtained for all sessions (Fig. 3), the differences were smaller at the end of the procedure, but regarding the temporal distribution, the peak of licks percentage remained lower in the THC pre-treated group, even in these final sessions. THC-pre-treated rats reached lower peaks of licks, but the animals in this group kept drinking longer during the FT interval. This explains why there were no differences in the overall number of licks between groups during the last sessions and why the areas under the curve were comparable. Analyzing the total set of licks, the rats of this group drank similar amounts of water but with a different temporal pattern. The time point at which the peak occurred was also shifted to the right—or delayed—in most of the sessions (Fig. 6 and Table 1), but this effect lessened as sessions went on—and was particularly diminished in the last sessions. On the other hand, acute THC administration after SID acquisition delayed the appearance of the peak in the SID inter-food interval (Fig. 7 and Table 2). The animals that had not received drug previously showed a 6-s delay in their peak time compared to control sessions, but with similar lick percentage levels at the highest point. However, the animals that had been pre-treated with THC previously showed a lower peak percentage of licks, but the difference about *when* this peak happened was less pronounced. Their temporal pattern was affected regarding the height of the curve that remained at a similar level from seconds 12 to 21, which represents a longer peak duration, but with lower kurtosis compared to the control group. This effect of licking for longer during the FT interval was already seen in the acquisition sessions. These results showed the way in which THC disrupts the temporal distribution pattern of SID, that is, decreasing and postponing the distinctive ‘*burst*’ of licks in this procedure (Falk 1971). This effect has already been demonstrated in humans, where THC induces overestimation of time (Lieving et al. 2006; Perez-Reyes et al. 1991; Sewell et al. 2013), mainly at short intervals (McDonald et al. 2003)—and it has also been shown in timing procedures in animals. In this regard, Han and Robinson (2001) studied the acute effect of cannabinoid agonists THC and WIN 55,212–2 on the peak procedure in rats; however, in contrast with our findings, they reported a reduction in the peak time. In another study, Crystal et al. (2003) also explored the acute effect of the cannabinoid agonist WIN 55,212–2 in a bisection timing task, resulting in a dose-related decrease in sensitivity to time. Therefore, it seems that THC induces alterations in time estimation, but these effects depend on the period evaluated, the pattern of drug administration (acute *vs* chronic), their residual effects and the nature of the task.

Both the SID phenomenon and maladaptive habits with excessive behaviour features are models of disorders related to the compulsive spectrum (Everitt et al. 2001; Gillan and Robbins 2014; Gillan et al. 2016; Moreno and Flores 2012). Cannabinoids influence habit or stimulus–response memory mediated by the dorsal striatum (Goodman and Packard 2015). One study, which employed different tasks involving habit and goal-directed learning processes, showed that reinforcer devaluation reduced the response slower in animals treated with THC (Nazzaro et al. 2012). Likewise, recent studies have also characterized habit formation in rats with the SID procedure (Gregory et al. 2015; Merchán et al. 2019). Given the behaviour repetitiveness produced by the SID procedure and its relation to habit-like behaviour, THC might be expected to facilitate the development of SID and to result in behaviour persistence during extinction. However, we did not find an incremental influence of the preceding chronic administration of THC on SID acquisition; quite the opposite, a clear impairment of rapid learning was observed. However, a persistence pattern in the temporal distribution of SID was seen only in animals pre-treated with THC, which kept licking longer during the inter-food interval. It may be that this reflects the habitual aspect of behaviour seen in persistent action. Furthermore, even though during the first extinction session (Fig. 5) the percentage of licks was higher in animals pre-treated with THC, the differences between groups were not sufficient to reach statistical significance, although they did come close. The slightly higher resistance to extinction of SID in THC-pre-treated animals might again reflect a habit-like behavioural characteristic.

Once SID had developed, we also evaluated the possible effects that acute THC administration could cause in subjects previously treated with this drug and in subjects that had no prior THC exposure. Our results showed that acute administration of a 5-mg/kg dose of THC decreased SID performance only in animals chronically pre-treated with THC, while in animals that had not been previously treated with the drug, SID was not affected. These results differ from those reported by Wayner et al. (1973a, b), who observed an increase in licks—but it should be noted that the THC doses employed were lower (1–3 mg/kg). This acute effect found only in the performance of pre-treated animals could be a sensitization-like effect, which leads to suggest that the prior experience with THC makes to develop a vulnerability to the effects of the drug after certain time without contact with it. Behavioural sensitization to THC effects was previously reported in animal studies (Cadoni et al. 2001), in which its subsequent single administration after a preceding prolonged exposure resulted in elevated locomotor activity, sniffing, gnawing and motor stereotypes. Furthermore, cross-sensitization effects were also reported with morphine (Cadoni et al. 2001) and nicotine (Panlilio et al. 2013), suggesting that THC can facilitate the use of other drugs of abuse. Our work provides support for sensitization effects derived from the consumption of cannabinoids and the propensity to potentiate their effects later.

THC is known to activate dopamine transmission through its action on the type 1 cannabinoid receptor (Laviolette and Grace 2006) that are co-localized with dopamine D2 receptor in GABAergic medium spiny neuron terminals, and cannabinoid agonists increased the interactions of these two types of receptors (Bagher et al. 2017). However, differential effects of THC on the dopamine system have been reported to depend on whether administration is acute or chronic. Acute THC administration increased dopamine release and neuron activity, whereas chronic THC administration altered dopamine D2/3 receptor signalling in nucleus accumbens and caudate/putamen (Bloomfield et al. 2016; Ginovart et al. 2012), and causes increased sensitivity to the presynaptic actions of dopamine D2 receptor agonists (Moreno et al. 2003). Several studies have evidenced the involvement of the dopaminergic system in SID. Both dopaminergic neuron lesions and the administration of dopamine antagonists—haloperidol, clozapine and pimozide—reduced already acquired SID and affected SID development (Didriksen et al. 1993; Mittleman et al. 1994; Mittleman and Valenstein 1986; Snodgrass and Allen 1989), whereas the dopamine D2/3 receptors agonist quinpirole increased this nonregulatory drinking behaviour in rats (Schepisi et al. 2014). It has been also demonstrated that high drinker rats showed higher dopamine D2 receptor binding than low drinkers in the SID procedure (Pellón et al. 2011). All these data together seem to suggest that alterations in the dopamine system may be involved in delayed SID acquisition after chronic THC administration.

Nonetheless, the reduction in licking observed after THC acute administration may also point to potential therapeutic use for compulsive behaviour. In this regard, marble burying in rodents—a behaviour which is also considered to reflect symptoms of OCD (Londei et al. 1998)—decreased after administration of different cannabinoids such as WIN 55,212–2 or cannabidiol (Gomes et al. 2011; Nardo et al. 2014). However, in order to clarify the therapeutic potential of THC in the SID procedure, future research is needed to ascertain the involvement of several relevant issues such as dose–response, sex and genetic background. Given the differences in dose–response effects of cannabinoids on behaviour (reviewed in Mechoulam and Parker 2013), it would be appropriate to test different chronic doses over time before SID acquisition. Moreover, several studies have demonstrated that female rats are more sensitive to cannabinoids than males (Fattore et al. 2007; 2010)—a finding that highlights the importance of the identification of sex-specific factors to guide the development of treatments more accurately. Finally, there is ample evidence that genetic background plays an important role in the individual vulnerability to psychiatric disorders (Adriani et al. 2003; Cadoni 2016; Driscoll 1982). Several different rat strains have shown deficits in inhibitory control responses, impulsivity or vulnerability to drug use related to the level of drinking on SID (Flores et al. 2014; Íbias and Pellón 2014). Moreover, Fisher 344 rats exhibit differences in the endocannabinoid system compared to Lewis rats (Brand et al. 2012; Coria et al. 2014; Rivera et al. 2013), which are considered an animal model for the study of genetic vulnerability to drug addiction (Cadoni 2016; Kosten and Ambrosio 2002). These two points lead us to hypothesize that genetic background could be a relevant variable in the effects of THC on SID.

In summary, the results of the experiment conducted show that prior chronic treatment with THC delays acquisition of adjunctive behaviour, confirming that cannabinoid consumption can disrupt learning, possibly causing alterations in time estimation. In addition, THC effects can be amplified later after an acute consumption reflecting a sensitization-like effect.

## Supplementary information

Below is the link to the electronic supplementary material.

Supplementary file1 (PDF 3554 KB)
