# Supplementary material for: Chronic ∆-9-tetrahydrocannabinol administration delays acquisition of schedule-induced drinking in rats and retains long-lasting effects
Source: Psychopharmacology (Berl). 2021 Aug 26;239(5):1359–72. doi: 10.1007/s00213-021-05952-2 (PMC9110535; doi:10.1007/s00213-021-05952-2)
Supplement: Supplementary file 1 — Supplementary file1 (PDF 3554 KB) [file 213_2021_5952_MOESM1_ESM.pdf]

Supplementary information for Psychopharmacology

Chronic Δ-9-Tetrahydrocannabinol administration delays acquisition of schedule-induced drinking in rats and retains long-lasting effects

Esmeralda Fuentes-Verdugo[1], Gabriela E. López-Tolsa[1,2], Ricardo Pellón[1], Miguel Miguéns[1]

1. Departamento de Psicología Básica I, Facultad de Psicología, Universidad Nacional de Educación a Distancia (UNED), C/ Juan del Rosal 10, Ciudad Universitaria, 28040-Madrid, Spain.
2. Facultad de Psicología, Universidad Nacional Autónoma de México (UNAM), Av. Universidad 3004, Col. Copilco – Universidad, 04510 Ciudad Universitaria, Ciudad de México, México.

Corresponding author:

Miguel Miguéns, Departamento de Psicología Básica I, Facultad de Psicología, Universidad Nacional de Educación a Distancia (UNED), C/ Juan del Rosal 10, Ciudad Universitaria, 28040-Madrid, Spain.

Email: mmiguens@psi.uned.es

Telephone: +34 913987971

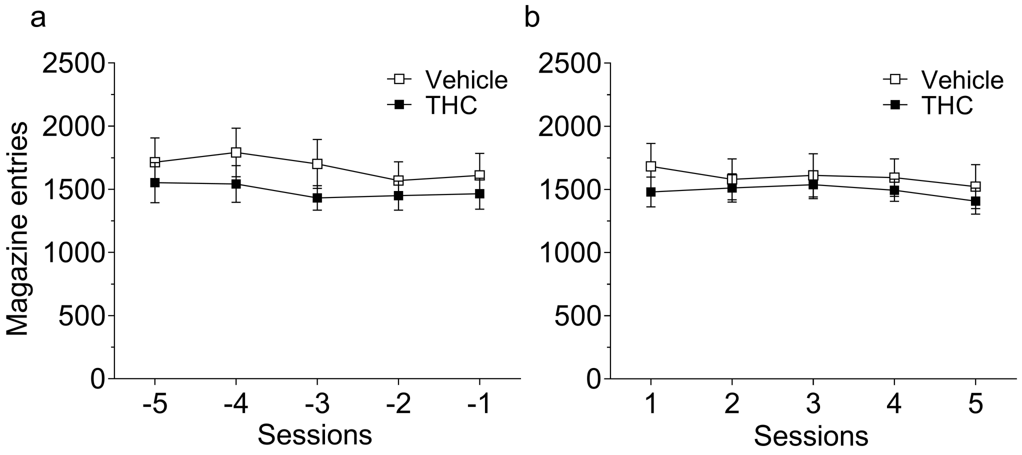

Fig. 1 Total number of magazine entries after SID acquisition and maintenance. The figure represents the total number of magazine entries (mean ± SEM) developed for vehicle (white squares, n=9) or THC pre-treated animals (black squares, n=9) during the last five acquisition sessions, established as the baseline to transform the data in acquisition and drug test phases (a), and during the five sessions conducted before extinction used as the baseline to transform the data of this phase (b).

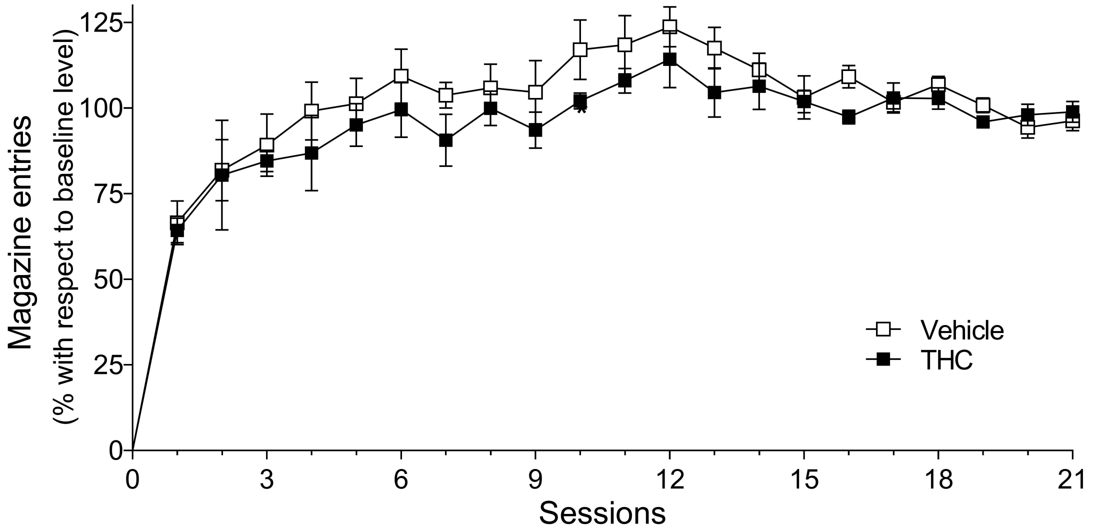

Fig. 2 Chronic effects of THC on magazine entries during SID acquisition. The figure represents the percentage of magazine entries with respect to the last five sessions average of the acquisition phase. The percentage of magazine entries is represented throughout 21 SID sessions (mean ± SEM). White squares represent vehicle pre-treated rats (n = 9), and the black ones THC (5 mg/kg) pre-treated rats (n = 9). ANOVA results: *session* ($F_{5,73}$=6.762; GG (ε) =0.227; p<0.0001; η^2_p = 0.297), *treatment x session* ($F_{5,73}$=0.492; p=0.764; η^2_p=0.03), *treatment* ($F_{1,16}$=0.207; p=0.655; η^2_p=0.013).

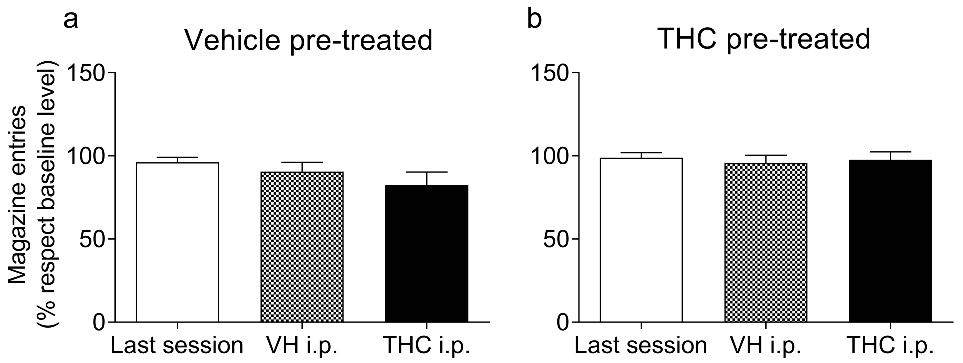

Fig 3. Acute effects of THC (5mg/kg i.p.) on magazine entries during SID procedure. The percentage of magazine entries with respect to the previous last five acquisition sessions (baseline level) is represented in the group pre-treated with vehicle (a) and the group pre-treated with THC (b). The data represent the mean ± SEM in the last session, the control session with a preceding vehicle i.p. injection, and the test session with a preceding THC i.p injection (n=9 in each group). ANOVA results: *session* ($F_{2,36}$=6.762; p=0.111; η^2_p = 0.115), *treatment x session* ($F_{2,36}$=1.402; p=0.259; η^2_p=0.072), *pre-treatment* ($F_{1,18}$=3.031; p=0.073; η^2_p=0.168).

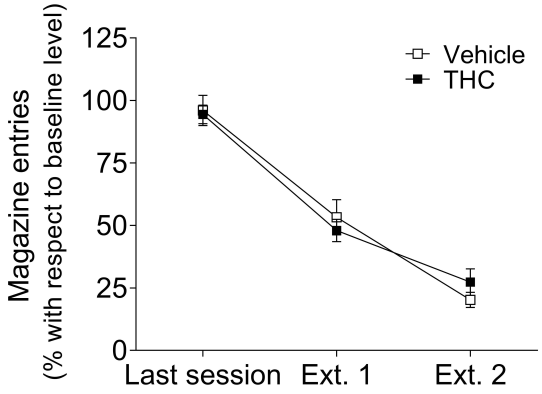

Fig 4 Chronic effects of THC on magazine entries during SID extinction. The figure represents the percentage of magazine entries with respect to baseline level (mean ± SEM) in the two SID extinction sessions for vehicle and THC pre-treated animals (n=9 in each group). ANOVA results: *session* ($F_{1,23}$=136.26; GG (ε) =0.647; p<0.0001; η^2_p = 0.883), *treatment x session* ($F_{1,23}$=2.038; p=0.164; η^2_p=0.102), *treatment* ($F_{1,18}$=0.649; p=0.431; η^2_p=0.035).

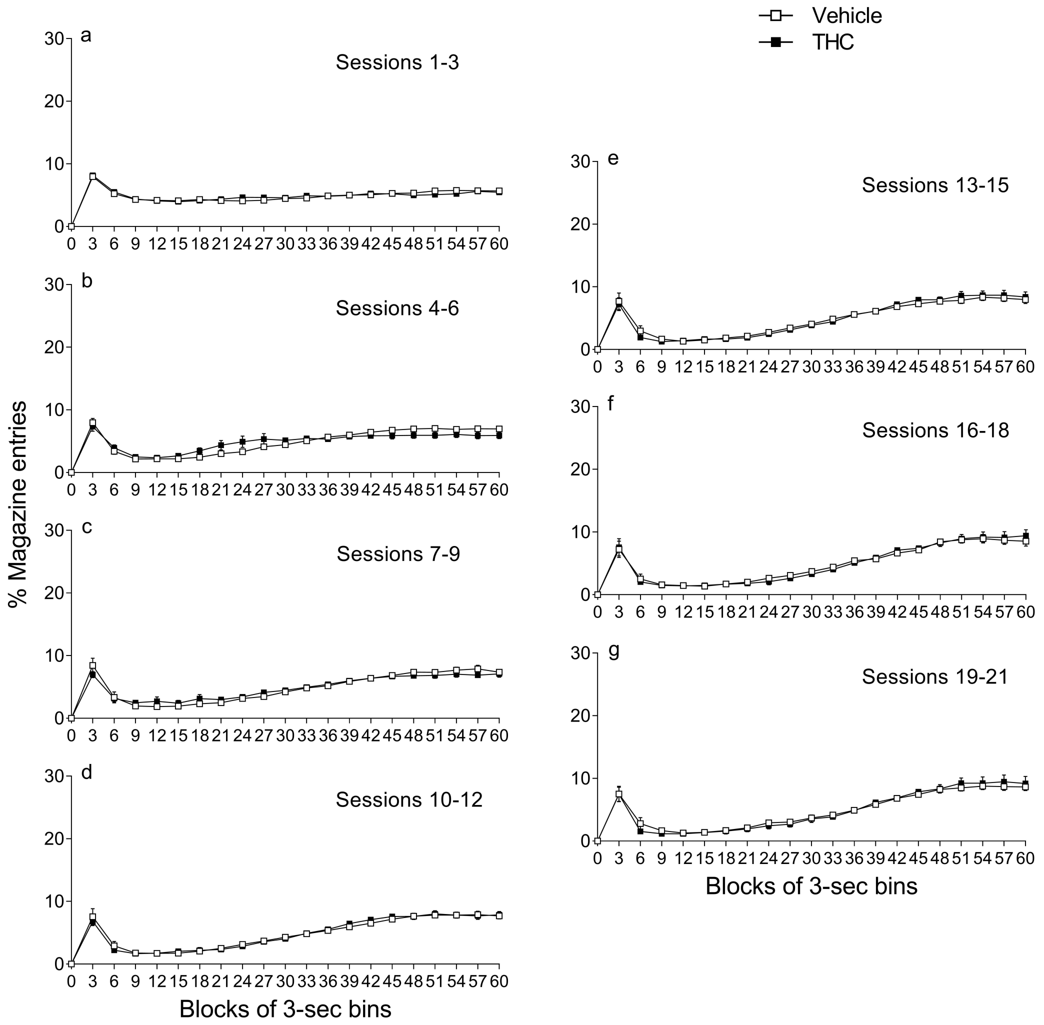

Fig 5 Temporal distribution of magazine entries throughout SID acquisition sessions. The figure displays the percentage of magazine entries with respect to the total number of entries performed in the inter-food interval for each rat (mean ± SEM) throughout successive 3-sec bins in animals pretreated with vehicle or THC (n=9 in each group). Separate sets of 3 sessions are presented to show acquisition development.

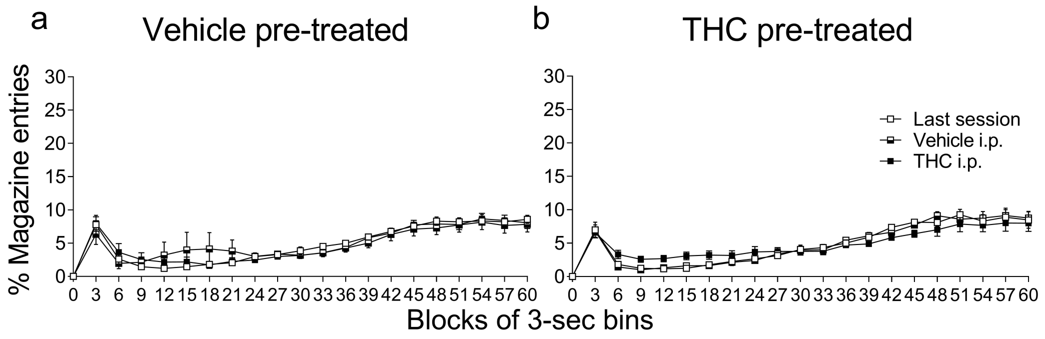

Fig 6 Acute effects of THC (5mg/kg i.p) in the temporal distribution of magazine entries in animals pretreated with THC or vehicle. Data show the percentage of licks with respect to the total number of licks performed in the inter-food interval (mean ± SEM) in animals pretreated with THC (a) or vehicle (b) throughout successive 3-sec bins of the 60 sec inter-food interval during the last acquisition session, the control session with a preceding vehicle i.p. injection, and the test session with a preceding THC i.p injection of 5 mg/kg (n=9 in each group).
